# Supplementary material for: Evaluation of COVID-19 related knowledge and preparedness in health professionals at selected health facilities in a resource-limited setting in Addis Ababa, Ethiopia
Source: PLoS One. 2021 Feb 10;16(2):e0244050. doi: 10.1371/journal.pone.0244050 (PMC7875347; doi:10.1371/journal.pone.0244050)
Supplement: S1 File — (PDF) [file pone.0244050.s001.pdf]

Health professional preparedness: Questionnaire

SECTION 1: GENERAL INFORMATION

1. Your age in years: …………..

2. Your gender: [1. Male [2. Female

3. The name of your hospital: …………………………………..

4. Your profession: [1. Doctor [2. Pharmacist [3. Nurse/Midwifery [4. Medical Laboratory Technologist/laboratory technician diploma

5. Your working department:

[1. Emergency Department [2. Intensive Care Unit [3. Outpatient Clinic/department

[4. Infectious Disease Department [5. Respiratory Department [6. Medical ward

[7. Laboratory unit [8. Surgical ward [9. Laboratory

[10. Other, specify: ……………….

6. Your working experience as a healthcare professional (doctor, nurse, pharmacist, laboratory etc.) in years: ....

SECTION 2: ASSESSMENT OF THE AWARENESS AND PREPAREDNESS OF HOSPITAL STAFF AGAINST THE NOVEL CORONAVIRUS 2019

7. Have you ever experienced any outbreak (e.g., SARS, MERS, bird flu, other respiratory infection outbreak, etc.) in the past? (Select all that apply)

[1. No [2. SARS [3. MERS [4. Bird flu [5. Other respiratory infection outbreak specify: ………………. ....

8. Has there been any confirmed human infection of 2019-nCoV in your country? (Select all that apply)

[1. No [2. Yes, in my country [3. Yes, in my city [4. Yes, in my hospital

9. Please choose the sources of information from which you gained knowledge about the 2019-nCoV outbreak? (Select all that apply)

[1. Media (newspaper, television, radio, etc.)

[2. Social network (Facebook, Twitter, blog, etc.)

[3. Academic Training Courses

[4. Colleagues

[5. Government Organization such as Ministry of Health

[6. Other, specify: …………….

10. Have you participated in any formal training course for dealing with the 2019-nCoV outbreak?

[1. Yes [2. No

11. Has your hospital taken measure to keep staff informed with contemporary information about coronavirus?

[1. Yes [2. No

12. Do patients coming to your hospital for routine health care get information about coronavirus?

[1. Yes [2. No

13. Which of the following are symptoms of the 2019-nCoV infection? (Select all that apply)

[1. Fever [2. Cough [3. Sneezing [4. Runny nose [5. Sore throat

[6. Shortness of breath [7. Pressure/ pain in the hest

[6. Joint/muscle pain [7. Red eyes [8. Rash [9. Diarrhea [10. May present without symptoms

14. Which of the following tests should be performed for the diagnosis of 2019-nCoV infection? (Select all that apply)

[1. Real-time polymerase chain reaction (PCR) with respiratory material (nasopharyngeal or oropharyngeal swab/ sputum/ endotracheal aspirate or bronchoalveolar lavage)

[2. Real-time PCR with serum sample

[3. Chest X-ray

[4. Serological tests

[5. Other, specify: ………………………….

15. Do you feel that you keep yourself up to date on the latest information about case definitions for 2019-nCoV infection?

[1. Yes [2. No

16. Is there a protocol of triage and isolation in your hospital for patients with symptoms suspected of 2019-nCoV infection?

[1. Yes [2. No [3. I don't know

17. Upon admission, which of the following should be considered to identify patients at risk of having 2019-nCoV infection? (Select all that apply)

[1. The presence of symptoms of diarrhea

[2. The presence of symptoms of a respiratory infection

[3. History of travel to areas experiencing transmission of 2019-nCoV

[4. History of contact with possible infected patients

18. In your hospital, is an Airborne Infection Isolation Room (AIIR) available?

[1. Yes [2. No [3. I don't know

19. If an Airborne Infection Isolation Room in your hospital is unavailable, do you know where to transfer a patient with suspected or confirmed 2019-nCoV infection?

[1. Yes [2. No

20. Do you consider yourself prepared for the management of the 2019-nCoV outbreak?

[1. Yes [2. No

21. Do you consider your hospital prepared for the management of the 2019-nCoV outbreak?

[1. Yes [2. No [3. I don't know

22. Please rate how satisfied you are with the current medical equipment in your hospital for the management of the 2019-nCoV outbreak:

[1. Very unsatisfied [2. Unsatisfied [3. Neutral [4. Satisfied [5. Very satisfied

23. In case of contact with possible 2019-nCoV patients, do you know how to use personal protective equipment (PPE)?

[1. Yes [2. No

24. In case of contact with confirmed 2019-nCoV patients, do you know how to perform isolation procedures on the patients to minimize chances for exposure?

[1. Yes [2. No

25. To what extent do you have confidence in handling suspected 2019-nCoV patients?

[1. Not at all [2. To a little extent [3. To some extent

[4. To a considerable extent [5. To a great extent

26. Has your hospital established procedures for controlling visitors to known or suspected 2019-nCoV patients?

[1. Yes [2. No [3. I don't know

27. Which of the following measures should be taken to prevent transmission from known or suspected 2019-nCoV patients? (Select all that apply)

[1. Frequently clean hands by using alcohol-based hand rub or soap and water

[2. Eat boiled and cooked food

[3. Put facemask on known or suspected patients

[4. Place known or suspected patients in adequately ventilated single rooms

[5. All health staff members wear protective clothing

[6. Avoid moving and transporting patients out of their area unless necessary

[7. Routinely clean and disinfect surfaces in contact with known or suspected patients

28. Do you know the precautionary measures to take when performing aerosol-generating procedures (such as tracheal intubation, non-invasive ventilation, tracheotomy, bronchoscopy, cardiopulmonary resuscitation, etc.) on 2019-nCoV patients?

[1. Yes [2. No

29. Do you know the criteria to guide evaluation of persons under investigation (PUI) for 2019-nCoV infection?

[1. Yes [2. No

30. Do you know where to take the report form and how to report a potential 2019-nCoV case or exposure to facility infection control leaders and public health officials?

[1. Yes [2. No

31. Do you know who to contact in a situation where there has been an unprotected exposure to a known or suspected 2019-nCoV patient?

[1. Yes [2. No

32. Do you know what to do if you have signs or symptoms suspected of 2019-nCoV infection?

[1. Yes [2. No

33. Do you know who to contact (chain of command) in outbreak situations in your hospital? Yes

[1. Yes [2. No

34. What suggestions might you have for improving the preparedness of health staff against 2019-nCoV in your hospital?

…………………………………………………………………………………………………

…………………………………………………………………………………………………

………………………………………………………………………………………

THANK YOU FOR TAKING THIS SURVEY.
