# Supplementary material for: Evaluation of COVID-19 related knowledge and preparedness in health professionals at selected health facilities in a resource-limited setting in Addis Ababa, Ethiopia
Source: PLoS One. 2021 Feb 10;16(2):e0244050. doi: 10.1371/journal.pone.0244050 (PMC7875347; doi:10.1371/journal.pone.0244050)
Supplement: S2 File — (DOCX) [file pone.0244050.s002.docx]

የጤናባለሙያዎችየኮቪድ 19 ዝግጁነትመጠይቅ

ክፍል 1: አጠቃላይመረጃ

| 1. ዕድሜ(በአመት) | ............................... |
| --- | --- |
| 1. ፆታ | 1. ወንድ 2. ሴት |
| 1. የሚሰሩበትሆስፒታልስም | …………………………….. |
| 1. ሙያዎ | 1. ዶክተር  2. ፋርማሲስት  3. ነርስ / አዋላጅ  4. የላቦራቶሪቴክኖሎጂስት / የላቦራቶሪቴክኒሽያን |
| 1. የሥራክፍልዎ | 1. የድንገተኛአደጋክፍል  2. የተጠናከረእንክብካቤክፍል  3. የተመላላሽታካሚክሊኒክ / መምሪያ  4. ተላላፊበሽታመምሪያ  5. የመተንፈሻአካላትህክምናክፍል  6. የጠቅላላህክምናክፍል  7. የላቦራቶሪክፍል  8. የቀዶጥገናክፍል  9. ላቦራቶሪ  10. ሌሎች፡ይግለፁ…………………. |
| 1. በጤናባለሙያነትየሥራልምድዎ (በአመት) | …………………………….. |

ክፍል 2፡ በኖቨልኮርኖቫይረስ2019 ዙሪያየጤናባለሙያዎችየግንዛቤእናዝግጁነትምዘና

| 1. ከዚህበፊትወረርሽኝአጋጥሞዎትያውቃል? (ለምሳሌሳርስ፣ መርስ፣የወፍጉንፋንእናሌላየመተንፈሻአካልበሽታ) (ከ1 በላይመምረጥይቻላል)፡፡ | 1. አያውቅም  2. ሳርስ  3. መርስ  4. የወፍጉንፋን  5. ሌላየመተንፈሻአካልበሽታ፤ ይግለፁ…………… |
| --- | --- |
| 1. በአገርዎውስጥ የ 2019-nCoV የተረጋገጠኢንፌክሽንተገኝቷል? (ከ1 በላይመምረጥይቻላል)፡፡ | 1. አልተገኘም 2. አዎበአገሬውስጥ 3. አዎ ፣ በከተማዬውስጥ 4. አዎበሆስፒታሌውስጥ |
| 1. eK ¢y=É-19 Ÿ¾ƒ—¨< ¾S[Í U”ßcS<; | 1. ሚዲያ (ጋዜጣ ፣ ቴሌቪዥን ፣ ሬዲዮ ፣ ወዘተ)  2. ማህበራዊአውታረ-መረብ (ፌስቡክ ፣ ትዊተር ፣ ብሎግ)  3. የአካዳሚክስልጠናኮርሶች  4. የሥራባልደረቦች  5. እንደጤናጥበቃሚኒስቴርያሉየመንግሥትአደረጃጀ  6. ሌላ ፣ ይግለጹ |
| 1. ከኮቪድ 19 ጋርየተገናኘማንኛውምመደበኛሥልጠናላይተሳትፈዋል | 1. አዎ  2. አይደለም |
| 1. የሚሰሩበትሆስፒታልስለኮሮናቫይረስወቅታዊመረጃዎችንሰራተኞችእንዲያውቁለማድረግእርምጃወስዷል? | 1. አዎ  2. አይደለም |
| 1. መደበኛየጤናእንክብካቤለማግኘትወደሆስፒታልዎየሚመጡህመምተኞችስለኮሮናቫይረስመረጃያገኛሉ? | 1. አዎ  2. አይደለም |
| 1. ከሚከተሉትውስጥየትኛው የ 2019-nCoV ኢንፌክሽንምልክቶችናቸው? (ከ1 በላይመምረጥይቻላል)፡፡ | 1. ትኩሳት 2. ሳል  3. በማስነጠስ 4. የአፍንጫፍሳሽ  5. የጉሮሮህመም6. የትንፋሽእጥረት  7. በደረትአካባቢግፊት / ህመም  8. የመገጣጠሚያ / የጡንቻህመም  9. ቀይዓይኖች10. ሰውነትላይሽፍታ  11. ተቅማጥ  12. ምንምምልክትላይኖረውይችላል |
| 1. ለ 2019-nCoV ኢንፌክሽንምርመራከሚከተሉትምርመራዎችውስጥየትኛውመከናወንአለበት? (ከ1 በላይመምረጥይቻላል)፡፡ | 1. ፒሲአር (ከአፍንጫወይምጉሮሮበተወሰደናሙና)  2. ፒሲአር (ከደምናሙና)  3. የደረትኤክስሬይ  4. ሴሮሎጂካልሙከራዎች  5. ሌላ ፣ ይግለጹ…………………. |
| 1. ከ2019-nCoV ኢንፌክሽንበተገናኘወቅታዊመረጃእንዳለዎትይሰማዎታል? | 1.አዎ  2. አይደለም |
| 1. የበሽታውምልክትላላቸውታካሚዎችበሆስፒታልዎውስጥለይቶማቆያፕሮቶኮልአለ? | 1. አዎ  2. አይደለም  3. አላውቅም |
| 1. በሽተኛሆስፒታልዎውስጥሲመጣ ለ2019-nCoV ኢንፌክሽንተጋላጭእንደሆነየሚጠቁሙምልክቶችየትኞቹናቸውብለውያስባሉ? | 1. የተቅማጥምልክቶችመኖር  2. የመተንፈሻአካላትችግርመኖር  3. የ 2019-nCoV ስርጭትንወደአለባቸውአካባቢዎችየጉዞታሪክ  4. በበሽታውከተያዙበሽተኞችጋርየመገናኘት(ንክኪ) ታሪክ |
| 1. በሆስፒታልዎውስጥbአየርወለድበሽታዎችየተያዙሰዎችንየማግለያክፍል (AIIR) ይገኛል? | 1. አዎ  2. አይደለም  3. አላውቅም |
| 1. በሆስፒታልዎውስጥbአየርወለድበሽታዎችየተያዙሰዎችንየማግለያክፍል(AIIR)ከሌለከ2019-nCoV ኢንፌክሽንየተጠረጠሩወይምመያዛቸውየተረጋገጠበሽተኞችወዴትመላክእንዳለብዎትያውቃሉ? | 1. አዎ  2. አላውቅም |
| 1. ለ2019-nCoV ወረርሽኝእራስዎንዝግጁነኝብለውያስባሉ? | 1.አዎ  2. አይደለም |
| 1. ለ2019-nCoV ወረርሽኝሆስፒታልዎዝግጁነውብለውያስባሉ? | 1.አዎ  2. አይደለም  3. አላውቅም |
| 1. የ 2019-nCoV ወረርሽኝንለመመርመርናለማከምበሆስፒታሉውስጥአሁንባለውየህክምናመሳሪያዎምንያህልእንደረካዎይገለፁ፡ | 1. በጣምረክቻለሁ 2. ረክቻለሁ 3. ገለልተኛ 4. አልረካሁም 5. በፍፁምአልረካሁም |
| 1. የ 2019-nCoV ህመምተኞችጋርንክኪበሚኖርበትጊዜየመከላከያግብአቶችን (PPE)እንዴትእንደሚጠቀሙያውቃሉ? | 1. አዎ  2. አላውቅም |
| 1. በ2019-nCoV ኢንፌክሽንመያዛቸውከተረጋገጠህመምተኞችጋርንክኪበሚፈጠርበትጊዜየመጋለጥእድልንለመቀነስበሕመምተኞችላይየመለየትሂደቶችእንዴትማከናወንእንደሚችሉያውቃሉ? | 1. አዎ  2. አላውቅም |
| 1. የተጠረጠሩ የ 2019-nCoV ህመምተኞችንለማስተናገድምንበራስዎላይያህልእምነትአለዎት? | 1. በጭራሽየለኝም 2. በጥቂቱ 3. በከፍተኛደረጃ |
| 1. በሆስፒታልዎየተጠረጠሩወይምየ2019-nCoVእንዳለባቸውየተረጋገጡበሽተኞችጠያቂዎችን(ጎብኚዎችን) ለመቆጣጠርየተዘረጋየአሰራርስርዓትአለ? | 1. አዎ  2. የለም  3. አላውቅም |
| 1. የ2019-nCoVኢንፌክሽንከተጠረጠሩወይምበሽታውእንዳለባቸውከተረጋገጡሰዎችወደሌሎችእንዳይተላለፍ   ለመከላከልከሚከተሉትእርምጃዎችውስጥየትኛውመወሰድአለበት(ከ1 በላይመምረጥይቻላል)፡፡ | 1. በሳኒታይዘርወይምበሳሙናናበውሃእጅንበተደጋጋሚማፅዳት 2. የበሰለምግብመመገብ 3. በበሽታውመያዛቸውየሚታወቁወይምየተጠረጠሩሕመምተኞችየፊትጭምብልማድረግ 4. በበሽታውመያዛቸውየሚታወቁወይምየተጠረጠሩሕመምተኞችንአየርበደንብየሚገባበትክፍልውስጥማድረግ 5. ሁሉምየጤናባለሙያዎችአስፈላጊውንየመከላከያግብአትመልበስ 6. አስፈላጊካልሆነበስተቀርታካሚዎችንከአካባቢያቸውእንዳይንቀሳቀሱእናእንዳይወጡማድረግ 7. በበሽታውመያዛቸውየሚታወቁወይምየተጠረጠሩሕመምተኞችንጋርንክኪያላቸውንቦታዎችበተደጋጋሚማፅዳት |
| 1. በ2019-nCoV ኢንፌክሽንየተያዙህመምተኞችላይኤሮሶል-የሚያመነጩሂደቶችን (እንደትራኪያልኢንቱቤሽን፣ ትራኪቶቶሚ ፣ ብሮንኮስኮፕ) በሚሰሩበትጊዜመውሰድያለብዎትንየጥንቃቄእርምጃዎችያውቃሉ? | 1. አዎ  2. አላውቅም |
| 1. በ2019-nCoV ኢንፌክሽንበምርመራላይያሉሰዎችን (PUI) ምዘናለመምራትመስፈርቶችን (PUI) ያውቃሉ? | 1. አዎ  2. አላውቅም |
| 1. የ2019-nCoV ጋርየተያያዘወይምየተጠረጠረህመምተኛሲገጥምዎትየሪፖርትፎርሙንየትሊገኝእንደሚችል፡ እንደሚወስዱእናእንዴትሪፖርትማድረግእንደሚችሉያውቃሉወይምለተቋሙየኢንፌክሽንቁጥጥርአመራሮችእናለሕዝብጤናባለሥልጣናትእንዴትእንደሚያሳውቁያውቃሉ? | 1. አዎ  2. አላውቅም |
| 1. የ2019-nCoVኢንፌክሽንከተጠረጠሩወይምበሽታውእንዳለባቸውከተረጋገጡሰዎችጥንቃቄየጎደለውተጋላጭነትያለበትሁኔታቢኖርምንንእነደሚያናግያውቃሉ? | 1. አዎ  2. አላውቅም |
| 1. የ2019-nCoV ኢንፌክሽንምልክቶችወይምምልክቶችካለብዎምንማድረግእንዳለብዎያውቃሉ? | 1. አዎ  2. አላውቅም |
| 1. በሆስፒታልዎውስጥየወረርሽኝሁኔታዎችበተመለከተማንንእንደሚያነጋግሩወይምየግንኙነትሰንሰለቱንያውቃሉ? | 1. አዎ  2. አላውቅም |
| 1. የ2019-nCoV ኢንፌክሽንጋርበተገናኘበሆስፒታልዎውስጥየጤናሰራተኞችንዝግጁነትለማሻሻልምንሀሳቦችአሉዎት? | ………………………………………………………………………………………….  ………………………………………………………………………………………….  ………………………………………………………………………………………….  …………………………………………………………………………………………. |
