# Supplementary material for: Evaluation of COVID-19 related knowledge and preparedness in health professionals at selected health facilities in a resource-limited setting in Addis Ababa, Ethiopia
Source: PLoS One. 2021 Feb 10;16(2):e0244050. doi: 10.1371/journal.pone.0244050 (PMC7875347; doi:10.1371/journal.pone.0244050)
Supplement: Attachment — Submitted filename: PONE-D-20-19732_reviewer 2.pdf [file pone.0244050.s003.pdf]

PLOS ONE

Evaluation of COVID-19 Related Health Professionals Knowledge and Preparedness in Selected Health Facilities in resource limited setting Addis Ababa, Ethiopia

--Manuscript Draft--

Manuscript Number:	PONE-D-20-19732
Article Type:	Research Article
Full Title:	Evaluation of COVID-19 Related Health Professionals Knowledge and Preparedness in Selected Health Facilities in resource limited setting Addis Ababa, Ethiopia
Short Title:	Assessment of health professionals knowledge and preparedness towards COVID-19
Corresponding Author:	Zelalem Desalegn<br>Addis Ababa University<br>Addis Ababa, ETHIOPIA
Keywords:	Health professional; Pandemic; COVID-19; knowledge; preparedness; Ethiopia
Abstract:	Background<br><br>WHO declared infection with SARS-CoV-2 as a pandemic. Experiences from SARS in 2003 have shown health professionals are at higher risk. Hence, periodic assessment of knowledge and preparedness is crucial in fight the pandemic.<br><br>Objectives<br><br>The research aimed at assessing awareness and preparedness of health professionals against COVID-19 among selected hospitals in Addis Ababa, Ethiopia.<br><br>Methods<br><br>A facility based cross sectional study was conducted between March and April,2020. A total of ten health facilities comprised of six government and four private hospitals were considered into the study. Higher proportion of health professionals at front line included taking into account their level of exposure for COVID-19. Data collection was done using a self-administered questionnaire. Data was entered using EpiData and analysis was done using SPSS software. A descriptive statistics and association were done using Chi-square test.<br><br>Results<br><br>A total of 1334 health professionals participated in the study. About fifty one percent were females and a higher proportion , 39.9% were nurses/midwifes followed by doctors, 29.8 %. Of these,29.8% had formal training on COVID-19. The participants mean knowledge score was 16.45 (±4.4). 58.7%, 26.5%, and 14.7% of healthcare professionals have moderate, good and had poor knowledge about COVID-19, respectively. Besides 63.2% indicated that they have been updated on COVID-19 from the hospital. 76.5% health professionals replied that television, radio and newspapers were primary sources of information. Interestingly, the finding demonstrated that health professionals were turned out to be prepared against COVID-19.<br><br>Conclusion<br><br>The finding showed that health professionals were moderately knowledgeable .Moreover, the assessment declared that they had good preparation towards the pandemic. However, further work on preventive measures is mandatory so as to combat the pandemic.
Order of Authors:	Zelalem Desalegn
	Negussie Deyessa
	Brhanu Teka

Powered by Editorial Manager® and ProduXion Manager® from Aries Systems Corporation

	Welelta Shiferaw
	Meron Yohannes
	Damen Hailemariam
	Adamu Addissie
	Abdulnasir Abagero
	Mirgissa Kaba
	Alem Abrha
	Workeabeba Abebe
	Berhanu Nega
	Wondimu Ayele
	Tewodros Haile
	Yirgu Gebrehiwot
	Wondwossen Amogne
	Eva Kantelhardt
	Tamrat Abebe
**Additional Information:**	
**Question**	**Response**
**Financial Disclosure**<br><br>Enter a financial disclosure statement that describes the sources of funding for the work included in this submission. Review the submission guidelines for detailed requirements. View published research articles from *PLOS ONE* for specific examples.<br><br>This statement is required for submission and **will appear in the published article** if the submission is accepted. Please make sure it is accurate.	ZD and TA received the award from AAU. Grant number: VPRTT/PY-403/2020.<br>The fund was secured from Addis Ababa University. www.aau.edu.et<br>We assure you that the funders had no role in study design, data collection and analysis, decision to publish, or preparation of the manuscript

**Unfunded studies**<br>Enter: *The author(s) received no specific funding for this work.*<br><br>**Funded studies**<br>Enter a statement with the following details:<br>• Initials of the authors who received each award<br>• Grant numbers awarded to each author<br>• The full name of each funder<br>• URL of each funder website<br>• Did the sponsors or funders play any role in the study design, data collection and analysis, decision to publish, or preparation of the manuscript?<br>• **NO** - Include this sentence at the end of your statement: *The funders had no role in study design, data collection and analysis, decision to publish, or preparation of the manuscript.*<br>• **YES** - Specify the role(s) played.<br><br>* typeset	
**Competing Interests**<br><br>Use the instructions below to enter a competing interest statement for this submission. On behalf of all authors, disclose any competing interests that could be perceived to bias this work—acknowledging all financial support and any other relevant financial or non-financial competing interests.<br><br>This statement **will appear in the published article** if the submission is accepted. Please make sure it is accurate. View published research articles from *PLOS ONE* for specific examples.	he authors have declared that no competing interests exist.

**NO authors have competing interests**<br><br>Enter: *The authors have declared that no competing interests exist.*<br><br>**Authors with competing interests**<br><br>Enter competing interest details beginning with this statement:<br><br>*I have read the journal's policy and the authors of this manuscript have the following competing interests: [insert competing interests here]*<br><br>* typeset	
**Ethics Statement**<br><br>Enter an ethics statement for this submission. This statement is required if the study involved:<br><br>• Human participants<br>• Human specimens or tissue<br>• Vertebrate animals or cephalopods<br>• Vertebrate embryos or tissues<br>• Field research<br><br>Write "N/A" if the submission does not require an ethics statement.<br><br>General guidance is provided below. Consult the submission guidelines for detailed instructions. **Make sure that all information entered here is included in the Methods section of the manuscript.**	The research was approved by Institutional Review Board(IRB) of College of Health Sciences.<br>Approval/protocol number: 012/20/DMIP

Powered by Editorial Manager® and ProduXion Manager® from Aries Systems Corporation

**Format for specific study types**<br><br>**Human Subject Research (involving human participants and/or tissue)**<br>• Give the name of the institutional review board or ethics committee that approved the study<br>• Include the approval number and/or a statement indicating approval of this research<br>• Indicate the form of consent obtained (written/oral) or the reason that consent was not obtained (e.g. the data were analyzed anonymously)<br><br>**Animal Research (involving vertebrate animals, embryos or tissues)**<br>• Provide the name of the Institutional Animal Care and Use Committee (IACUC) or other relevant ethics board that reviewed the study protocol, and indicate whether they approved this research or granted a formal waiver of ethical approval<br>• Include an approval number if one was obtained<br>• If the study involved *non-human primates*, add *additional details* about animal welfare and steps taken to ameliorate suffering<br>• If anesthesia, euthanasia, or any kind of animal sacrifice is part of the study, include briefly which substances and/or methods were applied<br><br>**Field Research**<br><br>Include the following details if this study involves the collection of plant, animal, or other materials from a natural setting:<br>• Field permit number<br>• Name of the institution or relevant body that granted permission	
**Data Availability**<br><br>Authors are required to make all data underlying the findings described fully available, without restriction, and from the time of publication. PLOS allows rare exceptions to address legal and ethical concerns. See the PLOS Data Policy and FAQ for detailed information.	Yes - all data are fully available without restriction

A Data Availability Statement describing where the data can be found is required at submission. Your answers to this question constitute the Data Availability Statement and **will be published in the article**, if accepted.<br><br>**Important:** Stating 'data available on request from the author' is not sufficient. If your data are only available upon request, select 'No' for the first question and explain your exceptional situation in the text box.<br><br>Do the authors confirm that all data underlying the findings described in their manuscript are fully available without restriction?	
**Describe where the data may be found in full sentences. If you are copying our sample text, replace any instances of XXX with the appropriate details.**<br><br>• If the data are **held or will be held in a public repository**, include URLs, accession numbers or DOIs. If this information will only be available after acceptance, indicate this by ticking the box below. For example: *All XXX files are available from the XXX database (accession number(s) XXX, XXX.)*.<br>• If the data are all contained **within the manuscript and/or Supporting Information files**, enter the following: *All relevant data are within the manuscript and its Supporting Information files.*<br>• If neither of these applies but you are able to provide **details of access elsewhere**, with or without limitations, please do so. For example:<br><br>*Data cannot be shared publicly because of [XXX]. Data are available from the XXX Institutional Data Access / Ethics Committee (contact via XXX) for researchers who meet the criteria for access to confidential data.*<br><br>*The data underlying the results presented in the study are available from (include the name of the third party*	All relevant data are within the manuscript and its Supporting Information files.

and contact information or URL).<br>• This text is appropriate if the data are owned by a third party and authors do not have permission to share the data.<br><br>* typeset	
Additional data availability information:	

Manuscript Click here to access/download;Manuscript;Manuscript HP.docx

Evaluation of COVID-19 Related Health Professionals Knowledge and Preparedness in Selected Health Facilities in resource limited setting Addis Ababa, Ethiopia

Running title: Assessment of health professionals knowledge and preparedness towards COVID-19

Zelalem Desalgne, MSc, Assistant professor, PhDc[1], Negussie Deyessa, MD, MPH, Associate professor, PhD[2], BrhanuTeka, MSc, PhDc[1],Welelta Shiferaw MSc, MPH[5], Meron Yohannes, MSc, PhDc[7], Damen Hailemariam MPH, PhD, Professor[2], Adamu Addissie MD, MPH, Associate professor, PhD[2], Abdulnasir Abagero MPH[2], Mirgissa Kaba MPH, Associate professor, PhD[2], Workeabeba Abebe MD, Associate professor[3],AlemAbrha MSC, PhD[10] ,Birhanu Nega MD, Associate professor[8],Wondimu Ayele MPH, PhDc, Assistant professor[9], Tewodros Haile MD, Associate professor[4], Yirgu Gebrehiwot MD, Associate professor[5], Wondwossen Amogne MD, Associate professor[6], Eva Kantelhardt MD, MPH, Professor[11], Tamrat Abebe MSc, PhD[1]

Affiliation of authors:

[1] Addis Ababa University, College of Health Sciences, School of Medicine, Department of Microbiology, Immunology and Parasitology

[2]Addis Ababa University, College of Health Sciences, School of Public Health

[3]Addis Ababa University, College of Health Sciences, School of Medicine, Department of Pediatrics

[4] Addis Ababa University, College of Health Sciences, School of Medicine, Department of Internal Medicine

[5] Addis Ababa University, College of Health Sciences, School of Medicine, Department of Psychiatry

[6]Addis Ababa University, College of Health Sciences Clinical Director of Health Sciences, School of Medicine, Department of Internal Medicine

[7]Addis Ababa University, College of Health Sciences, School of Allied Health Sciences, Department of Medical Laboratory Technology

[8]Addis Ababa University, College of Health Sciences School of Medicine, Department of Surgery

[9]Addis Ababa University, College of Health Sciences , School of Public Health

[10] Yekatit-12 Hospital Medical College Addis Ababa, Ethiopia

[11]Medical Faculty, Martin-Luther-University, Halle-Wittenberg, Halle (Saale), Germany

* **Corresponding author**: Zelalem Desalegne

Addis Ababa University, College of Health Sciences, School of Medicine, Department of Microbiology, Immunology and Parasitology

Present Address: Phone no: +251912029567

P.Box: 9086, Addis Ababa, Ethiopia:E-mail: tzollove@gmail.com; zelalem.desalegn@aau.edu.et

Key words: Health professional, Pandemic, COVID-19, knowledge, preparedness, Ethiopia

Abstract

Background: WHO declared infection with SARS-CoV-2 as a pandemic. Experiences from SARS in 2003 have shown health professionals are at higher risk. Hence, periodic assessment of knowledge and preparedness is crucial in fight the pandemic.

Objectives: The research aimed at assessing awareness and preparedness of health professionals against COVID-19 among selected hospitals in Addis Ababa, Ethiopia.

Methods: A facility based cross sectional study was conducted between March and April,2020. A total of ten health facilities comprised of six government and four private hospitals were considered into the study. Higher proportion of health professionals at front line included taking into account their level of exposure for COVID-19. Data collection was done using a self-administered questionnaire. Data was entered using EpiData and analysis was done using SPSS software. A descriptive statistics and association were done using Chi-square test.

Results: A total of 1334 health professionals participated in the study. About fifty one percent were females and a higher proportion, 39.9% were nurses/midwifes followed by doctors, 29.8 %. Of these,29.8% had formal training on COVID-19. The participants mean knowledge score was 16.45 (±4.4). 58.7%, 26.5%, and 14.7% of healthcare professionals have moderate, good and

had poor knowledge about COVID-19, respectively. Besides 63.2% indicated that they have been updated on COVID-19 from the hospital. 76.5% health professionals replied that television, radio and newspapers were primary sources of information. Interestingly, the finding demonstrated that health professionals were turned out to be prepared against COVID-19.

Conclusion: The finding showed that health professionals were moderately knowledgeable .Moreover, the assessment declared that they had good preparation towards the pandemic. However, further work on preventive measures is mandatory so as to combat the pandemic

Key words: Health professional, Pandemic, COVID-19, knowledge, preparedness, Ethiopia

Introduction

The rapid spread of Coronavirus Disease 2019 (COVID-19) worldwide has raised concerns around the world. Since the first case was detected in Wuhan City, China, the disease has spread rapidly. The pathogen identified as a cause of COVID-19 is currently called severe acute respiratory syndrome corona virus-2 (SARS-CoV-2) [1] that has a phylogenetic resemblance to SARS-COV-1 [2].With the dramatic increase in daily-confirmed global cases of COVID-19, the World Health Organization (WHO) has declared as a global pandemic on March 12, 2020 [3]. SARS-CoV-2spreads by human-to-human transmission through the droplet, feco-oral, and direct contact and has an incubation period of 2-14 days [4].

Healthcare workers (HCWs) are at a higher risk of having COVID-19. According to the experience of the 2003 SARS outbreak, one-fifth of the global burden of SARS cases were healthcare workers [5]. A number of risk factors have been identified during that time including lack of knowledge and preparedness as well as poor infection control measures, lack of training,

and poor compliance with the use of PPE while in contact with patients suspected or not and high-risk procedures

In the current pandemic, as of 21 April 2020 countries reported to WHO that over 35, 000 health workers were infected with COVID19 [6]. In support with established facts, further investigations on the aerodynamic nature of the virus revealed differences in the concentrations of SARS-CoV-2 RNA aerosols in different areas of two hospitals of Wuhan [2]. The areas with high load were those prone to a crowd with carriers of the virus. Thus, healthcare workers are expected to be at high risk of infection. Hazards include pathogen exposure, long working hours, psychological distress, fatigue, occupational burnout, stigma, and physical and psychological violence.

One can recognize that the transmission of the COVID-19 among HCWs is associated with overcrowding, absence of isolation room facilities, and environmental contamination. However, this is likely compounded by the fact that some HCWs have inadequate knowledge of infection prevention practices [7]. From different previous studies, the knowledge and attitudes of medical staff towards infectious diseases and their willingness to work during an epidemic have been explored including the knowledge and attitudes of critical care clinicians during the 2009 H1N1 influenza pandemic [8, 10].

Protection of HCWs and prevention of intra-hospital transmission of infection are important aspects in epidemic response and this requires that HCWs must have updated knowledge regarding the source, transmission, symptoms and preventive measures of COVID-19. Lack of knowledge and misunderstandings among HCWs lead to delay in diagnosis, the spread of the disease, and poor infection control practices. Therefore, it is well understood knowledge of a disease can influence HCWs’ attitudes and practices, and incorrect attitudes and practices

directly increase the risk of infection[11]. Hence, KAP survey provide a suitable format to evaluate existing programs and to identify effective strategies for behavior change in society and helps to predict outcomes of planned behavior.

For this purpose, the WHO, CDC and other organizations are providing several guidelines and started online courses and training sessions to raise knowledge and preparedness regarding prevention and control ofCOVID-19. Currently, there is scarce information regarding the knowledge and preparedness level of HCWs in Ethiopia. Therefore, the aim of this study is to assess knowledge and preparedness among HCWs in Addis Ababa, Ethiopia.

.

Methods

Study Design and study population

We used a cross-sectional surveys to assess health professionals knowledge and preparedness in selected health facilities from March to April 2020, Addis Ababa Ethiopia. A total of 1334 participants involved in the study. The participants were composed of medical doctors, nurses/midwives, pharmacists, and medical laboratory technologists/technicians.

Statistical power and sample size

A single population proportion formula was used with the assumption of 50% of health professionals have knowledge and preparedness for an epidemic including COVID-19 and its management, with a 4% margin of error at a 95% confidence level, with a design effect of 2.0, and adding 15% for non-response which gave final sample size of 1373.

Sampling procedure

For this study, a multistage sampling was in place. The first stage was stratifying health facilities by governmental and private ownership, and the second stage was cluster sampling among the two groups, taking a list of facilities of each stratum. Six government and four private hospitals were included in the study through random selection. All health providers included to the study conveniently.

Data collection

A standardized self-administered questionnaire adopted from published protocol [12] was modified and used. The questionnaire has two sections, a section with general information asking

about age, sex, profession, department, and the hospital where they belong and year of work experience as a health professional. The second section focus on COVID-19 related knowledge and preparedness of the participants and the respective hospital preparedness and practices of working.

The knowledge and preparedness assessment focuses on personal and institutional issues. Personal issues include knowledge about signs & symptoms, identification of persons at risk, prevention measures and tests recommended to confirm exposure of SARS-CoV-2. Besides, the questions address issues if the health professionals workers are prepared on personal level , if they know how to use PPE, what to do if exposed or develop signs and symptoms, and if they have knowledge on case management. At institutional level, the questionnaire address if there is any triage protocol, isolation room, required equipment for case management if the need comes, risky medical procedures that generate aerosols and if chain of command is in place.

knowledge Scoring system

By considering the total marks for each category, the score is grades as poor, moderate, or good based on cut-offs done based on modified Bloom cutoff point as follows: $\leq$ 12= poor, 13-19= moderate and $\geq$20=good

Data processing and Analysis

The data obtained were entered using EpiDataVersion 3.1and analyzed using SPSS soft ware version 25.Descriptive statistics was applied to calculate frequencies and proportions. Chi-square test was used to investigate association among variables.

Ethical consideration

Institutional Review Board (IRB) of the College of Health Sciences of the Addis Ababa University approved the study protocol (Protocol number: 012/20/DMIP). Written informed consent was obtained from all participants. Participation of respondents was voluntary.

Results

Socio-Demographic characteristics

The study included 1334 health professionals whose age ranged 18 to 59 years, with a mean age of 30.71 ± 6.19 50.7% of the participants were females. 39.9% of the participants were nurses/midwifes, followed by doctors 29.8%. Table 1 shows the demographic characteristics of the study participants.

Table 1 Demographic characteristics of health professionals

Demographic characters		Number	Percent
**Sex**	Male	656	49.3
	Female	675	50.7
**Age group**	Mean age, 30.71 ± 6.19<br>≤24	116	8.7
	25-29	546	40.9
	30-34	374	28.0
	35-39	142	10.6
	≥40	108	8.1
**Profession**	Doctor	397	29.8
	Pharmacist	193	14.5
	Nurse/midwife	532	39.9
	Medical laboratory	207	15.5
**Hospitals**	Government Hospitals	802	60.1
	Private Hospitals	532	39.9
**Service year of employment (in**	≤5	791	59.3
	>5	456	34.2

years)

Knowledge towards COVID-19

The detail of the response given by the health professionals is shown in Table 2. The assessment tool of knowledge considered a total of 25 item in the are of signs and symptoms of COVID-19, diagnostic test that should performed for COVID-19, conditions upon admission required to identify patients at risk of having COVID-19, and approaches to prevent transmission of COVID-19 in hospitals.

Based on the assessment, over 80% of health professionals identified the correct response. About 73% identified RT-PCR using respiratory samples as a diagnostic test for SARS-Co2-infection. With regard to identification of patients at risk of having COVID-19 upon hospital admission, 88.5% and 76 % identified travel to COVID-19 affected area and contact with infected person, respectively. Frequent use of hand washing with soap and water/alcohol-based hand rubs and putting facemask on known or suspected patients were identified by 93.4 % and 91% of health professionals, respectively. The mean knowledge score was 16.45±4.4 (range 2-25). Of the total, 58.7%, 26.5% and 14.7% had moderate, good and poor knowledge on COVID-19 , respectively.

With respect to prevention of transmission measures from known or suspected patients, health care professionals knew most of the preventive measures. Majority of the health professionals 1251(93.8%) responded that hand washing with soap and water and hand rubbing with alcohol could be the possible way for the prevention of COVID-19. Interestingly, 1011(75.8%) of them assumed that eating cooked and boiled food is protective in fight against COVID-19. 1214(91%)

of participants responded that putting mask on suspected or known patients prevent transmission of the SARS-cov-2.

Table 2 Health professionals knowledge on signs, diagnostic methods, identification criteria and prevention measures towards COVID-19

	Number	Percent
**K1: Sign and symptoms for COVID-19**		
i. Fever	1304	97.8
ii. Cough	1278	95.8
iii. Sneezing	977	73.2
iv. Runny nose	658	49.3
v. Sore throat	1124	84.3
vi. Shortness of breath	1096	82.2
vii. Pressure/pain in the chest	486	36.4
viii. Joint/muscle pain	583	43.7
ix. Red eyes	237	17.8
x. Rash	166	12.4
xi. Diarrhea	493	37.0
xii. May present without symptoms	394	29.5
**K2: COVID-19 diagnostics**		
i. RT-PCR with respiratory samples	974	73.0
ii. RT-PCR with serum samples	768	57.6
iii. Chest X-ray	501	37.6
iv. Serological tests	401	30.1
**K4: Identification criteria for patients at risk of COVID-19**		
i. Presence of diarrhea	435	32.6
ii. Respiratory infection symptoms	986	73.9
iii. Travel to COVID-affected areas	1181	88.5
iv. Contact with possible infected patients	1014	76.0
**K5: Measures for prevention of transmission from known or suspected patients**		
i. Frequent washing with soap and water/alcohol based hand rub	1251	93.8
ii. Avoiding eating uncooked food	1011	75.8
iii. Putting facemask	1214	91.0
iv. Placing known or suspected patients in adequately ventilated single rooms	1161	87.0
v. Wearing protective clothing	1144	85.8
vi. Avoiding moving and transporting patients	1105	82.8
vii. Routine cleaning and disinfecting surfaces	1075	80.6

Interestingly, the study explored knowledge level across profession in those with and without formal training. Those with formal training had moderate knowledge accounted doctors 95(69.3%), nurses/midwifes 106(69.3), 34(64.2%) and Medical laboratory 36(65.5%). However, those without the training slightly lower moderate knowledge levels. As shown in Table 3, the study assessed the association of knowledge of health professionals with demographic characteristics.

Table 3 Association of knowledge with demographic characteristics

Characteristics		Knowledge/knowledge scores			p-value
		**Poor N (%)**	**Moderate N (%)**	**Good N (%)**	
**Sex**	Male	79 (12.1)	366 (55.9)	210 (32.1)	< 0.001
	Female	117 (17.3)	415 (61.5)	143 (21.2)	
**Age group**	≤24	21 (18.1)	75 (64.7)	20 (17.2)	0.003
	25-29	89 (16.3)	312 (57.2)	144 (26.4)	
	30-34	44 (11.8)	234 (62.6)	96 (25.7)	
	35-39	22 (15.5)	70 (49.3)	50 (35.2)	
	≥40	6 (5.6)	72 (66.7)	30 (27.8)	
**Profession**	Doctor	18 (4.5)	214 (54.0)	164 (41.4)	< 0.001
	Pharmacist	48 (24.9)	111 (57.5)	34 (17.6)	
	Nurse/midwife	98 (18.4)	333 (62.6)	101 (19.0)	
	Medical laboratory	31 (15.0)	123 (59.4)	53 (25.6)	

Preparedness of health professionals towards the pandemic

The details assessment of preparedness of health professionals and the respective hospital they belong is shown in Table4. The assessment of preparedness of health professionals has shown mixed results, less than 50 % health professionals know precaution measures during risky procedures, where to report a potential case or exposure or the criteria that guides evaluation of

persons under investigation. Among the total, 220(56.8%) doctors, 127(67.2%) pharmacists, 323(61.5%) nurses/midwifes and 119(58.6%) medical laboratory professionals responded that as they were prepared for COVID-19 management (P-value < 0.10) . Out of the total, 287(73.6%) doctors, 110(57.3%) pharmacist, 303(57.5%) nurses/midwifes and 131(63.9%) of them were confident enough on how to use PPE in case of possible contact with COVID-19 patient s(P-value < 0.001). The finding revealed that majority of them were too little extent confident in handling suspected COVID- 19 patients (P-value < 0.07).

Assessment of the respective hospital's preparedness by the health professionals

With regard to the preparedness of the respective hospitals, approximately 50% health professionals think that their respective hospital is prepared against COVID-19(P-value <0.001). The study demonstrated that closely 50% were unsatisfied with available medical equipment against COVID-19 in their hospitals (P-value < 0.002). However, 57.4% indicated their hospital have established protocol for triage.

COVID-19 related source of information

Of the total, Media (television, radio and newspapers) were the primary source of information for most of the health care professionals 1020 (76.5%) followed by Social network (Facebook, twitter, blog) accounting 899(67.4%). Among the total, 398 (29.8%) have got a formal training regarding COVID outbreak and 843 (63.2%) responded their hospital took measures to keep staff updated about COVID-19.

Discussion

The success in the fight against COVID -19 is dependent in the involvement of the public, the healthcare workers and the appropriate actions made by government. As the public is informed to stay at home, the HCW go to their clinics and hospitals. Experience from China and Italy tells over 3,300 and 20% of HCW got infected respectively and some died [13].Those who were not infected or survived suffered from physical and mental exhaustions. Alongside their personal safety concerns, HCWs are worried to their families as well as patients.

Despite its public health burden and economic impact, there is an ample of knowledge deficits among health professionals. Unless efforts incurred to improving the knowledge of the health professionals and maximizing their preparedness, the pandemic would have tremendous impact on health care system and consequently would alter COVID-19 prevention and management. At times like this, assessing the awareness and preparedness of the health professionals is helpful to identify gaps and correct on timely.

Previously, it has been shown that age and service year are an important factor which could place an individual at higher risk for an infection. Our finding indicated that majority of the participants were in age range 25-29 years and served for < 5 years which was in agreement with a study from Nigeria. Such characteristics were explained with being a major source of infection and a means for spreading of infection [14].

Globally, a wide ranges of differences in the knowledge and preparedness level towards COVID-19. However, knowledge in many ways could impact attitude, behaviors and individuals' level of positive attitude, which consequently change in behavior in a broader context [11]. Our finding demonstrated that two-third of the participants (58.7%) had moderate awareness/knowledge. The overall knowledge of the participated HCW regarding signs & symptoms, identification of persons at risk of developing diseases, case definition of COVID-19, appropriate tests offered to

suspected cases as wells as high-risk patients, preventive measures that help to minimize the risk of transmission of SARS-CoV-2 was good. But, 40-60% of the HCWs did not know whom to contact in the hospital outbreak situation, upon unprotected exposure, criteria to guide evaluation of suspects, and how to perform isolation procedures. Besides, 55% of the HCWs did not know the precautionary measures when performing aerosol generating procedures despite claiming they had the latest information.

In line with our study, a study from Iran indicated that more than half of the health-professional (56.5%) had good knowledge about sources, transmission, symptoms, signs, prognosis, treatment, and mortality rate of COVID-19[15]. In contrary, another study from Iran revealed that HCWs have insufficient knowledge about COVID-19 but showed positive perceptions of the prevention of COVID-19 transmission [16].

Our study finding was by far lower than a study done elsewhere. A study from China demonstrated that a higher proportion (89%) of HCWs had sufficient knowledge of COVID-19. In their study, they have indicated that doctors showed higher knowledge scores (38.56 ± 3.31) than nurses (37.85 ± 2.63) and paramedics (36.72 ± 4.82) [17].Moreover, our study was lower than the study from China where it showed the participants possessing sufficient knowledge were 88.4% [18].

The study explored the overall mean knowledge score. Accordingly, the mean knowledge score was 16.45±4.4 (range 2-25) . Our finding was in line with the overall nurses knowledge score accounted 16.35 ± 3.3 which has been reported from Iran [15].The recent finding was by far higher than a finding from Vietnam which reported a mean score of knowledge of 8.17±1.3. Their finding showed that HCWs had a high level of knowledge and a positive attitude towards the COVID-19 outbreak[18].However, much work has to be done to better mitigate the challenge

and keep the safety of health professionals. Therefore, health professionals should be provided with latest training in all aspects of COVID-19 including prevention modalities, transmission mode, diagnostic strategies, prevention strategy, management of cases, what to do during exposure and command of chain where to report unusual events so as to contain the pandemic.

In general, having sufficient knowledge may reflect the successful dissemination of information about COVID-19 by different media. In this regard, the study explored from where the health professionals were getting health related information on COVID-19. Accordingly, media (television, radio and newspapers) were the primary source of information followed by social network (facebook, twitter, blog). This could be explained by high rate of transmission of COVID-19 in the world which might have increased the health professionals attention and subsequent knowledge of this pandemic. In agreement with our findings, a study from Iran indicated a stunning figure accounting 60% of HCWs used social media as a source of information [16].

With this respect, another finding indicated that the sources of information for the nurses were the World Health Organization and the Ministry of Health (55.3%), social applications (48.23%), and media (42.35%) [15], which are somehow credible and reliable source of information unlike the others. Comparably, a study from Vietnam demonstrated that the main sources of COVID-19 information were social media and the Ministry of Health website 91.1% and 82.6%, respectively[18]. In our study non identified the national guidelines or the WHO websites as a source of information which is major gap that requires immediate attention.

The wide spread use of the Internet and its availability to wider sectors of society has made it a major source of information. Though information from social media has great positive impact on

prevention and control of the disease, it needs some kind of regulation to minimize/avoid wrong information so as to combat the current situation in the right manner.

In our finding, we were able to assess the knowledge of health professionals about the possible diagnostic modalities for COVID-19. With this regard, the findings proved that the majority of the participants were aware of the potential samples to be collected and the respective method for COVID-19 diagnosis. This is interesting and encouraging because this will have an impact on early detection, management of patients and prevention and control of the pandemic. Considering the dynamics of the pandemic, we should work aggressively in providing up-to-date information on the type of samples, procedures of appropriate sample collection, the principle of the methods, the limitation and interpretation of the finding.

By now, what we have at hand to fight against COVID-19 is that firmly applying infection prevention protocol at all levels. With respect to knowledge on preventive measures from known or suspected patients, 93.8% of the health professionals mentioned hand washing with soap and water and hand rubbing with alcohol as an important prevention measures against COVID-19 . Since the primary means of containing the pandemic is through prevention, having such understanding is critical because such measure could possibly break the transmission of SARS-cov-2. But, health professionals should be provided with latest information on infection prevention and control (IPC).

Additionally, one of the most important aspects of an outbreak is identification of patients who have symptoms and are at a great risk of having the disease. With this regard, the assessment showed that the majority of healthcare professionals recognized identification criteria of patients of having COVID 19. Basically, such understanding enables the health professionals identify cases at the early stage so as to establish appropriate management and minimize the spread of

SARS-cov-2 infection among. With a continuous support from the concerned stakeholders, the health professionals would combat the current pandemic and any possible outbreak in the future.

This baseline study is limited by its cross-sectional design but the study will serve as a guide for planning and implementing interventions targeted at controlling possible epidemic.

Conclusion

The current study showed that the health professionals had moderate knowledge towards the pandemic. The health professionals preparedness was encouraging in many aspects towards COVID-19. However, improving their knowledge and preparedness is mandatory to cope up with the pandemic.

Acknowledgements: We would like to thank all the study participants and the data collectors. Our appreciation goes to Addis Ababa University and College of Health Sciences management for the all the support.

References

[1]. Guan W, Ni Z, Hu Y, Liang W, Ou C, He J, et al. Clinical Characteristics of Coronavirus Disease 2019 in China. *N Engl J Med*. 2020; 1–13. doi:10.1056/nejmoa2002032

[2]. Lu R, Zhao X, Li J, Niu P, Yang B, Wu H, et al. Genomic characterisation and epidemiology of 2019 novel coronavirus: implications for virus origins and receptor binding. Lancet. 2020;395: 565–574. doi:10.1016/S0140-6736(20)30251-8

[3]. Quan-xinLong ,Hai-jun Deng, Juan Chen, Jie-li Hu , Bei-zhong Liu, Pu Liao et al. Antibody responses to SARS-CoV-2 in COVID-19 patients: the perspective application of serological tests in clinical practice. https://doi.org/10.1101/2020.03.18.20038018

[4]. World Health Organization. Fact sheet. Calls for healthy, safe and decent working conditions for all health workers, amidst COVID-19 pandemic.https://www.who.int/news-room/detail/28-04-2020-who-calls-for-healthy-safe-and-decent-working-conditions-for-all-health-workers-amidst-covid-19-pandemic (Accessed on 28 April, 2020)

[5]. World Health Organization.2003.Fact sheet.

[6]. World Health Organization. Novel coronavirus (COVID-19) situation. Available online:https://https://experience.arcgis.com/experience/685d0ace521648f8a5beeeee1b9125cd (Accessed on 30April 2020)

[7]. Wu Z, McGoogan JM. Characteristics of and Important Lessons From the Coronavirus Disease 2019 (COVID-19) Outbreak in China: Summary of a Report of 72 314 Cases From the Chinese Center for Disease Control and Prevention [published online ahead of print, 2020 Feb 24]. *JAMA*. 2020;10.1001/jama.2020.2648. doi:10.1001/jama.2020.2648

[8]. Mehrdad Askarian, Mary-Louise McLaws, and MarysiaMeylan. Knowledge, attitude, and practices related to standard precautions of surgeons and physicians in university-affiliated hospitals of Shiraz, Iran. *Int J Infect Dis*. 2007 ;11(3): 213–219

[9]. Hamed Sarani, Abbas Balouchi, NosratollahMasinaeinezhad, and Ebrahim Ebrahimitabs.Knowledge, Attitude and Practice of Nurses about Standard Precautions for Hospital-Acquired Infection in Teaching Hospitals Affiliated to Zabol University of Medical Sciences (2014). *Glob J Health Sci*. 2016; 8(3): 193–198

[10]. Xiaochun Ma, Zhenyang He, Yushan Wang, Li Jiang, Yuan Xu, ChuanyunQian et al .Knowledge and attitudes of healthcare workers in Chinese intensive care units regarding 2009 H1N1 influenza pandemic.*BMC Infectious Diseases*.2011;11: 24

[11]. McEachan R, Taylor N, Harrison R, Lawton R, Gardner 2, Conner M. Meta-Analysis of the Reasoned Action Approach (RAA) to Understanding Health Behaviors. *AnnBehav Med*.2016;50(4): 592-612

[12]. Ahmad Taysir ,SzeJiaNg, AbdelrahmanGamil, LuuNgocMai, ShyamPrakash, NguyenLam et al. Knowledge and Preparedness of Hospital Staff against Novel Coronavirus: A Global Survey. DOI: 10.2139/ssrn.3550294

[13]. The Lancet. COVID-19: protecting health-care workers. *Lancet*. 2020;395(10228):922. doi:10.1016/S0140-6736(20)30644-9

[14]. Samuel Anu Olowookere, Emmanuel Akintunde Abioye-Kuteyi, Olusegun KayodeAdepoju, OluwaseunTaiwoEsan, Temitope Michael Adeolu et al.Knowledge, Attitude, and Practice ofHealth Workers in a Tertiary Hospital in Ile-Ife,Nigeria, towards Ebola Viral Disease. *Hindawi Publishing Corporation Journal of Tropical Medicine*.2015; 2015: 1-6 .http://dx.doi.org/10.1155/2015/431317

[15]. Nemati M, Ebrahimi B, Nemati F. Assessment of Iranian Nurse's knowledge and anxiety toward COVID-19 during the current outbreak in Iran. *Arch Clin Infect Dis*. Online ahead of print; 15(COVID-19); e102848.doi:10.5812/archid.102848

[16]. Akshaya Srikanth Bhagavathula, Wafa Ali Aldhaleei, Jamal Rahmani, Mohammad javad Ashrafi Mahabadi, Deepak Kumar Bandari .Novel Coronavirus (COVID-19) Knowledge and Perceptions: A Survey of Healthcare Workers.doi: https://doi.org/10.1101/2020.03.09.20033381

[17]. Zhou M, Tang F, Wang Y, Nie H, Zhang L, You G, Zhang M. Knowledge, attitude and practice regarding COVID-19 among health care workers in Henan, China.*Journal of Hospital Infection*, https://doi.org/10.1016/j.jhin.2020.04.012

[18]. Huynh G, Nguyen TNH, Tran VK, Vo KN, Vo VT, Pham LA. Knowledge and attitude toward COVID-19 among healthcare workers at District 2 Hospital, Ho Chi Minh City.*Asian Pac J Trop Med*2020:13.doi: 10.4103/1995-7645.280396

Supplementary data: Supplementary data to this article can be made available upon request.
