# Supplementary material for: Evaluation of COVID-19 related knowledge and preparedness in health professionals at selected health facilities in a resource-limited setting in Addis Ababa, Ethiopia
Source: PLoS One. 2021 Feb 10;16(2):e0244050. doi: 10.1371/journal.pone.0244050 (PMC7875347; doi:10.1371/journal.pone.0244050)
Supplement: Attachment — Submitted filename: Response to reviewers.docx [file pone.0244050.s004.docx]

**Response to reviewers**

**Manuscript number**: PONE-D-20-19732

**Manuscript title:** Evaluation of COVID-19 related knowledge and preparedness in health professionals at selected health facilities in a resource-limited setting in Addis Ababa, Ethiopia

**Dear editor and the reviewer,**

We are very grateful for the valuable comments and scientific guidance forwarded from the editor, Plos One journal team member and the respective reviewers. We have learnt a lot in the process of the revision process which would have great impact in the personal and professional development. Dear academic editor and reviewers, your inputs were highly critical in enriching the scientific paper to utmost standard.

Dear reviewers, we appreciate your commitment taken of reviewing the manuscript aimed exploring the response of health professionals toward COVID-19. Your comments were well taken and incorporated accordingly in the revised version of the manuscript.

To the most possible extent, we tried to entertain the entire forwarded comments. We have gone through the document and the respective additions, modification, elaboration and correction made whenever the need arise as per the comments concern. If there are anything to which the editor and the reviewers are not satisfied/ and or clear with, the authors are highly eager and open-minded to entertain the missed points at any time point.

Therefore, herewith please find enclosed the point-by-point reviewers and editor response. In the online submission, we uploaded documents including [1. Manuscript without track changes [2. Manuscript with track changes [3. Reviewer response [4. Supplementary material 1 [5. Supplementary material 2 [6. others as per the requirements

**With kind regards,**

Zelalem Desalegn Woldesonbet

**Submitting author,**

**Editor Comments**

1. Authors need to disclose the information on the first manuscript submitted to the

journal which is related to COVID-19 infection in the same local context.

Well taken. Based on the comment, we have considered research based information from the local context considered in the manuscript. This has happened in the introduction part and while discussing our findings. We appreciate your comment.

2. Grammatical errors throughout the manuscript and lack of clarity in most of the

paragraphs require extensive language editing.

Well taken. The grammatical errors and lack of clarity addressed in the revised version of the manuscript. Additionally, the revised version has been sent out for language proof-reading service and corrections were accommodated in the revised version of the manuscript. However, if there are any unaddressed, the authors are highly interested and ready to correct them as per the requirements and the scientific standard.

3. The main claims of the paper are focused on knowledge of healthcare workers and their reported preparedness of health care facilities to manage COVID-19 infection. Authors need to add more description on how significant are those claims for the improvement of service delivery and program operations at health facilities in the Introduction by citing appropriate literature.

Well taken. Accordingly, we have provided data demonstrating the relevance of doing research related with the current topic in the improvement of service delivery and program operations at health facilities.

4. Importantly, authors need to follow Strobe Guidelines for Observational Studies in the

write up of methods section and discussion. Limitations of the study are missing.

Well taken. STROBE guidelines for any cross-sectional study considered in the method section

5. It is critical to note that the conclusion section is weak and requires a revision.

Well taken. The conclusion section re-considered and written based the finding of the research finding. The change has been shown in the revised manuscript with track changes.

6. Please include a copy of Table 4 which you refer to in your text on page 19.

We appreciate your comments. We have included the copy of Table 4 in the text.

**Journal Requirements:**

When submitting your revision, we need you to address these additionalrequirements.

7. Please ensure that your manuscript meets PLOS ONE's style requirements, including

those for file naming.

Well taken. The authors believe that our manuscript meets the PLOS ONE's style requirements.

However, the authors are eager to address them with your directions if there are any unnoticed points in the revised version.

8. Please include additional information regarding the survey or questionnaire used in the study and ensure that you have provided sufficient details that others could replicatethe analyses. For instance, if you developed a questionnaire as part of this study and it is not under a copyright more restrictive than CC-BY, please include a copy, in both the original language and English, as Supporting Information.

Well taken. We have included the data collection instrument.

9. PLOS requires an ORCID iD for the corresponding author in Editorial Manager on

papers submitted after December 6th, 2016. Please ensure that you have an ORCID iD

and that it is validated in Editorial Manager.

Well taken. The submitting author has created the iD and validated in the Editorial manager

**Reviewer Comments to the Author**

*Dear Reviewers,*

The authors appreciate the very positive feed backs of the reviewers forwarded to the very relevant research questions. Our heartfelt thanks goes to the reviewers for their additional inputs and specific comments raised at different section of the manuscript.

**Reviewer #1**: A timely manuscript evaluating the knowledge and preparedness ofHCWs in Addis.

some minor comments

1- Recent work on KAPs of HCWs in Africa and beyond has been published. Authors

should search for these papers and cite them as appropriate.

Well taken. Research works which have been conducted in African context and beyond searched for the purpose to revealing the real picture and strengthening the manuscript further. A very recent research findings were considered in the introduction section for outlining what has been known so far in the area of the research problem. Additionally, we have used plenty of research findings while comparing our finding in the discussion section.

2. Method. Remove the number of participants from the study design (keep it only in the

results section)

Well taken. The number of participants removed from the study design section.

3. Use recent data to describe the global and local burden of COVID-19

We appreciate the comment. Well taken. Since an ample of researches based evidences are coming out and figures are changing from time to time, we accept of considering very latest data to describe COVID 19 in the local and global context and accommodated accordingly..

**Reviewer #2:**The corrections are done in the manuscript. The author should check the

manuscript and effect all the noted corrections.

1-The author should effects the Minor revisions and return the manuscript for finalevaluation and publication.

Well taken. Every given comments checked in the manuscript and considered in the revised version of the manuscript

2-It is important the author consult the work done by Ogolodom et al as recommended. Itwill help them seriously.

Well taken. On top of considering additional research data released at the local and global level, we were able to use the recommended research work done by Ogolodom et al. As you have mentioned, we found the paper very helpful for our purpose and used in the introduction section and discussion part of the revised manuscript.
