# Supplementary material for: Evaluation of COVID-19 related knowledge and preparedness in health professionals at selected health facilities in a resource-limited setting in Addis Ababa, Ethiopia
Source: PLoS One. 2021 Feb 10;16(2):e0244050. doi: 10.1371/journal.pone.0244050 (PMC7875347; doi:10.1371/journal.pone.0244050)
Supplement: Attachment — Submitted filename: R2R.docx [file pone.0244050.s005.docx]

**Date: October 16, 2020**

**To**:	**Dr. Joerg Heber**

Editor-in-Chief, PLOS ONE

**Subject**: **Submission 2nd revised version**

Dear Dr. Joerg Heber,

I am submitting the revised version of a manuscript entitled “Evaluation of COVID-19 Related Health Professionals Knowledge and Preparedness in Selected Health Facilities in resource limited setting Addis Ababa, Ethiopia”. At this critical time of pandemic, studies in COVID-19 are highly compulsory to be shared with in the global community and most especially if findings are published in highly visible journals like PLOSE ONE. It is obvious that the global society is currently fighting with the pandemic and researchers are trying their best to explore about it. Currently, there is scarce information regarding the knowledge and preparedness level of Health care workers about COVID-19 in Ethiopia. With this in mind, we conducted the research work and come up with an important finding.

I believe that PLOSE ONE is the most appropriate journal to share our results with the international scientific community as it invites multidisciplinary and interdisciplinary research works; hence, our manuscript perfectly fits with this aim and scope of the journal. Our results will guide the Ministry of Health, other stakeholders and programmers in Ethiopia where there is a scarcity of data about this global pandemic.

I confirm that this is an original article and not part of any published manuscript. There are no conflicts of interest to disclose and all the authors have contributed in many ways to the matured manuscript and finally approved the submission of the manuscript to your journal.

We appreciate for taking your precious time and forwarded your valuable comments. We considered all the comments. With this letter we kindly would like inform you that we uploaded new cover letter, clean manuscript without track change, revised manuscript with track changes.

We look forward receiving your feed back

Sincerely,

Zelalem Desalegne

Addis Ababa University

E-mail: [zelalem.desalegn@aau.edu.et](mailto:zelalem.desalegn@aau.edu.et)  ;[tzollove@gmail.com](mailto:tzollove@gmail.com);
