# Supplementary material for: Evaluation of COVID-19 related knowledge and preparedness in health professionals at selected health facilities in a resource-limited setting in Addis Ababa, Ethiopia
Source: PLoS One. 2021 Feb 10;16(2):e0244050. doi: 10.1371/journal.pone.0244050 (PMC7875347; doi:10.1371/journal.pone.0244050)
Supplement: Attachment — Submitted filename: Response to reviewers.docx [file pone.0244050.s006.docx]

**Response to reviewers**

**Manuscript number**: PONE-D-20-19732

**Manuscript title:** Evaluation of COVID-19 related knowledge and preparedness in health professionals at selected health facilities in a resource-limited setting in Addis Ababa, Ethiopia

**Dear editor and the reviewer,**

We are very grateful for the valuable comments and scientific guidance forwarded from the editor, Plos One journal team member and the respective reviewers. We have learnt a lot in the process of the revision process which would have great impact in the personal and professional development. Dear academic editor and reviewers, your inputs were highly critical in enriching the scientific paper to utmost standard.

Dear reviewers, we appreciate your commitment taken of reviewing the manuscript aimed exploring the response of health professionals toward COVID-19. Your comments were well taken and incorporated accordingly in the revised version of the manuscript.

We have gone through the document and the respective additions, modification, elaboration and correction made whenever the need arise as per the comments concern. If there are anything to which the editor and the reviewers are not satisfied/ and or clear with, the authors are highly eager and open-minded to entertain the missed points at any time point.

In the online submission, we uploaded documents including [1. Manuscript without track changes [2. Manuscript with track changes [3. Reviewer response [4. Proof reading service

**With kind regards,**

Zelalem Desalegn Woldesonbet

**Submitting author,**

**Reviewers and editor comments**

**Reviewer #1**: The authors have sufficiently addressed all comments by the reviewer. The manuscript is now acceptable in its current form. Well done !

**Reviewer #2**: I am okay with all the corrections done by the author.

**Editor Comments (if provided):**

**1. Authors have adequately addressed the comments of reviewers.**

We are grateful for the scientific inputs given by the academic editor and reviewers as well.

**2. Authors need to follow the journal guidelines in preparing the Reference section.**

Well taken and we used a Vancouver style as outlined in the ICMJE sample references ( National Library of Medicine) and considering previously published articles in Plos One journal to meet the journal requirements(Please refer line number 449 to 539).

**3. Still, there is a need to correct some grammatical errors to improve readability and to meet the journal standard.**

Well taken. In addition to the professional proof reading service, the authors have thoroughly gone through the revised manuscript to improve the readability and meeting the journal standard.

**Abstract:**

**1. LINE 69: "The current study revealed that" (to replace shows)**

Well taken. The stated phrase incorporated directly into the revised manuscript(Please refer line number 68).

Text

**LINE 122: "Despite the extensive efforts made so far, accumulated evidence indicates that" (to remove shows)**

We appreciate the comment. As per the comment" shows: replaced with the word " indicates ( please refer line number 121).

**LINE 160: "considering a KAP survey as a suitable format'**

Well taken. In the main document " is" replaced with " as" (please refer line number 150)

**LINE 157: " A total of six government hospitals and four private hospitals were included in the study**

The comment incorporated into the revised version (Please refer line number 156 to 158)

**" LINE 162: "A written informed consent"**

Well taken and incorporated in the revised version ( Please refer line number 163).

**LINE 168: "health professionals had knowledge and preparedness**

Thank you. The comment incorporated into the revised version of the manuscript ( Please refer line number 169).

**" LINE 169: "considering for a design effect of"**

We appreciate. The comment considered in the document (Please refer line number 170 to 1710).

**LINE 218: "The study included 1,334 health professionals"**

Thank you. We have considered the comment in the manuscript ( Please refer line number 171 to 172)

**LINE 229: "The finding showed that"**

Well taken. We have made the change ( Please refer line number 229).

**LINE 253: "represented doctors**

Well taken (Please refer line number 253).

**" LINE 302: please correct as follows- "that there were reported deaths of over 3,300 (20%) HCWs [22]."**

Yes, we accept the way you have constructed the description (Please refer line number 298 to 299).

**LINE 304: "HCWs were worried"**

We accepted the comment. Kindly edited in the revised version of the manuscript (Please refer line number 300)

**LINES 308-309: Please correct as follows- "At this time of crisis" rather than "At a time like this".**

Well taken and incorporated into the document (Please refer line number 306).

**LINE 319: "Our findings demonstrated that"**

Well taken. The comment incorporated into the revised version (Please refer line number 316).

**LINE 417: "which were somehow"**

We are grateful. The change considered in the revised manuscript (Please refer line number 414).

**LINE 426: "there should be a regulation"**

Well taken ( Please refer line number 423 to 424).

**LINE 432: "participants are recruited to the study"**

Well taken with the respective change in the document (Please refer line number 429).

**LINE 433: "where people had plenty of access"**

We appreciate the comment and considered in the document (Please refer line number 430)

**LINE 434: "consequently, the findings only reflected a sneak peak"**

Well taken and indicated in the manuscript (Please refer line number 431 to 432)

**LINE 438: "This study concluded that"**

Well taken and indicated in the manuscript ( Please refer line number 435).
